# Nanoreporter PET predicts the efficacy of anti-cancer nanotherapy

**DOI:** 10.1038/ncomms11838

**Published:** 2016-06-20

**Authors:** Carlos Pérez-Medina, Dalya Abdel-Atti, Jun Tang, Yiming Zhao, Zahi A. Fayad, Jason S. Lewis, Willem J. M. Mulder, Thomas Reiner

**Affiliations:** 1 Translational and Molecular Imaging Institute, Icahn School of Medicine at Mount Sinai, New York, New York 10029, USA; 2 Centro de Investigación en Red de Enfermedades Respiratorias, 28029 Madrid, Spain; 3 Advanced Imaging Unit, Centro Nacional de Investigaciones Cardiovasculares, CNIC, 28029 Madrid, Spain; 4 Memorial Sloan Kettering Cancer Center, Department of Radiology, New York, New York 10065, USA; 5 Department of Radiology, Weill Cornell Medical College, New York, New York 10065, USA; 6 Molecular Pharmacology & Chemistry Program, Memorial Sloan Kettering Cancer Center, New York, New York 10065, USA; 7 Department of Medical Biochemistry, Academic Medical Center, 1105 AZ Amsterdam, The Netherlands

## Abstract

The application of nanoparticle drug formulations, such as nanoliposomal doxorubicin (Doxil), is increasingly integrated in clinical cancer care. Despite nanomedicine's remarkable potential and growth over the last three decades, its clinical benefits for cancer patients vary. Here we report a non-invasive quantitative positron emission tomography (PET) nanoreporter technology that is predictive of therapeutic outcome in individual subjects. In a breast cancer mouse model, we demonstrate that co-injecting Doxil and a Zirconium-89 nanoreporter (^89^Zr-NRep) allows precise doxorubicin (DOX) quantification. Importantly, ^89^Zr-NRep uptake also correlates with other types of nanoparticles' tumour accumulation. ^89^Zr-NRep PET imaging reveals remarkable accumulation heterogeneity independent of tumour size. We subsequently demonstrate that mice with >25 mg kg^−1^ DOX accumulation in tumours had significantly better growth inhibition and enhanced survival. This non-invasive imaging tool may be developed into a robust inclusion criterion for patients amenable to nanotherapy.

## Abstract (web summary)

Nanoparticle drug formulations are currently used as cancer treatment but the response in patients is highly variable. Here, the authors developed a Zirconium-89 nanoreporter able to predict using PET, therapeutic accumulation and efficacy of anti-cancer nanoparticle drug formulations when co-injected in a murine breast cancer model.

## Graphical abstract

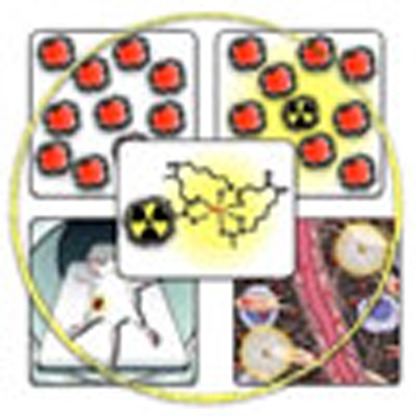

Clinically approved nanoparticle drug formulations such as Doxil or Abraxane are used to treat a wide range of cancers, including ovarian cancer, breast cancer and lung cancer^1^,^2^,^3^,^4^,^5^,^6^,^7^,^8^,^9^. Nanotherapies' numerous benefits include enhanced pharmacokinetics, increased drug stability and improved tumour bioavailability^10^,^11^,^12^. The variability of individual patient response to nanotherapy treatment remains a topic of concern and is believed to result from tumour permeability and drug clearance heterogeneity^4^. Identifying patients amenable to anti-cancer nanotherapy should therefore be based on individualized inclusion criteria derived from quantifiable procedures. Non-invasive imaging can aid this process^13^,^14^, but current clinical protocols lack specificity, while most experimental imaging-facilitated nanotherapy assessment studies have little translational potential. In response to this need, researchers have proposed labelling nanoparticle drug formulations for imaging-facilitated delivery^1^,^13^,^14^,^15^,^16^. A variety of (super)paramagnetic labels are available for magnetic resonance imaging (MRI), but generally require much higher concentrations for imaging, potentially compromising their utility as non-therapeutic imaging drugs^1^,^14^. Radioisotopes can be used to non-invasively visualize nanoparticle tumour delivery by nuclear imaging, and are generally applied in microdoses, orders of magnitude lower than needed to elicit a therapeutic response^14^. Unfortunately, such approaches require the chemical modification of a nanotherapy, which potentially compromises its functionality, rendering translation and clinical implementation far too expensive for general use. We believe an easy-to-prepare nanoreporter that can be co-injected with the clinically approved anti-cancer nanotherapy can overcome these issues. We here present a highly sensitive and accurate positron emission tomography (PET) liposomal nanoreporter for Doxil, and demonstrate its ability to predict therapeutic outcome based on tumour uptake in a mouse breast cancer model. Additionally, we show that this nanoreporter can be used in combination with other nanoformulations as well.

## Results

### Nanoreporter ^89^Zr-NRep and Doxil are physiochemically similar

**Figure 1:**
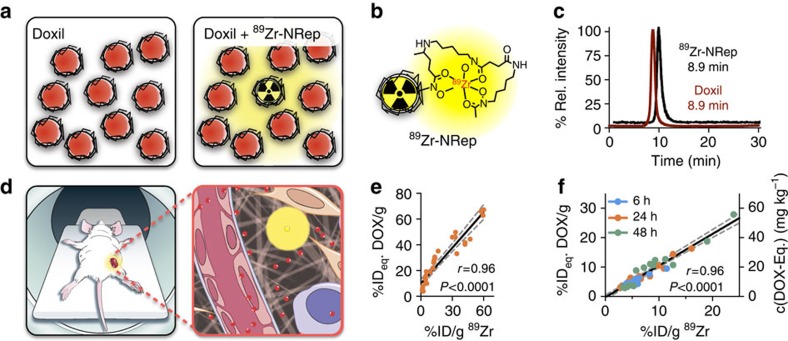
Nanoreporter PET imaging concept. (**a**) Schematic representation of the FDA-approved Doxil nanoformulation (left) and the ^89^Zr-NRep doped Doxil nanoformulation used in this study (right). (**b**) The liposomal nanoreporter ^89^Zr-NRep modified with ^89^Zr-chelating DFO. (**c**) Compared size exclusion retention times for clinical grade Doxil nanoformulation (fluorescence emission, red) and ^89^Zr-NRep (HPLC γ-counter, black). (**d**) Co-injecting ^89^Zr-NRep and Doxil allows non-invasive quantification of DOX delivery. © 2016, Memorial Sloan Kettering Cancer Center. (**e**) Correlation between ^89^Zr-NRep (%ID per g) and DOX (%ID_eq._/g) in blood. Data points (*N*=28) represent individual blood samples, from which activity was counted (γ-counter) before DOX was extracted and quantified. (**f**) Correlation between ^89^Zr-NRep (%ID per g) and DOX (%ID_eq._/g) uptake. Data points (*N*=45) represent tumour tissue from mice euthanized at 6 h (blue), 24 h (orange) or 48 h (green) post administration. Tissues were excised, and associated activity counted (γ-counter), before DOX was extracted from the tissues. Pearson's *r* coefficients were calculated to determine correlation.

Our Doxil nanoreporter ^89^Zr-NRep (Fig. 1a) consists of a pegylated liposome (Fig. 1b) labelled with ^89^Zr through a desferrioxamine B (DFO) functionalized phospholipid. The exact characteristics, composition and radiolabelling efficiency data are included in the Supplementary Information. ^89^Zr-NRep's size exclusion chromatography retention time is identical to Doxil (Fig. 1c), and as per dynamic light scattering size measurements, its size and Zeta potential, 100 nm and −20 mV respectively, are very similar to Doxil's (Supplementary Table 1).

### ^89^Zr-NRep uptake correlates with DOX tumour accumulation

Using a well-established mouse breast cancer model, we tested whether ^89^Zr-NRep's tumour radioactivity would report on Doxil tumour accumulation as quantified by non-invasive PET imaging (Fig. 1d). To validate our nanoreporter method, we first established its function *ex vivo*. On intravenous co-injection of a therapeutic dose of Doxil (20 mg kg^−1^) and ^89^Zr-NRep (1.0 mCi kg^−1^) in mice (*N*=6), we found a strong correlation between doxorubicin (DOX) and ^89^Zr in circulation (*r*=0.96, *P*<0.0001 (Pearson), Fig. 1e). Directly after injection of 20 mg kg^−1^ Doxil and 8 mCi kg^−1 89^Zr-NRep, blood levels remain higher than 90 mg kg^−1^ and 15 mCi kg^−1^ for DOX and ^89^Zr, respectively, over 24 h (Supplementary Fig. 1). *Ex vivo* radioactivity and DOX content in tumours was quantified at 6, 24 and 48 h after administration (*N*=24). We observed a strong correlation (*r*=0.96, *P*<0.0001 (Pearson)) between the DOX and ^89^Zr-NRep percentages of injected dose per gram tissue (%ID per g; Fig. 1f and Supplementary Fig. 2), which were determined in digested tumour tissue by spectrofluorimetry and gamma counting. Importantly, the near one slope of this correlation signifies that %ID per g of ^89^Zr equals %ID per g DOX, thereby indicating that the ratio of Doxil to ^89^Zr-NRep at time of injection remains the same after tumour accumulation.

### ^89^Zr-NRep PET allows quantifying DOX tumour accumulation

**Figure 2:**
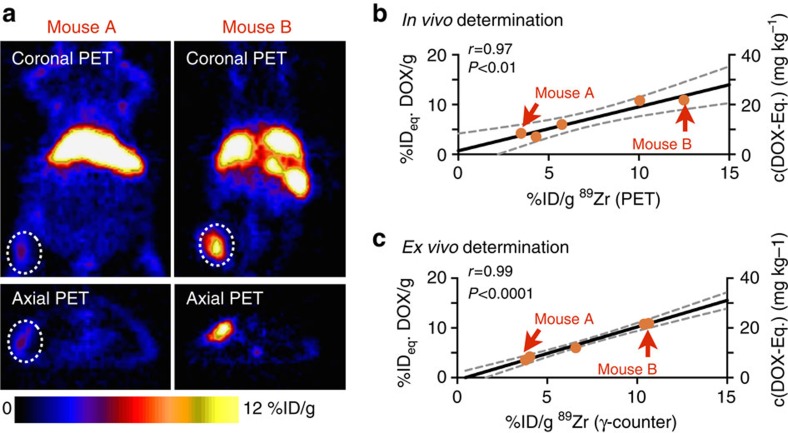
Non-invasive nanoreporter PET quantifies DOX tumour uptake. (**a**) Representative images of 4T1 tumour-bearing mice with low ^89^Zr-NRep uptake (mouse A, left) and high ^89^Zr-NRep uptake (mouse B, right). (**b**) Correlation of uptake values generated by non-invasive PET imaging and DOX tumour concentrations and (**c**) correlation of tumour-associated activity (measured *ex vivo*, γ-counter) and DOX tumour concentrations (*N*=5). Mice were injected with ^89^Zr-NRep/Doxil (0.14 mCi ^89^Zr-NRep, 10 mg kg^−1^ Doxil), underwent PET imaging at 24 h and were then killed to quantify tumour-associated activity (γ-counter) and DOX. (**a**) Shows representative mouse PET scans, and panels **b** and **c** show the corresponding obtained values. Pearson's *r* coefficients were calculated to determine correlation.

Next, we used non-invasive PET imaging to quantify DOX tumour accumulation. Tumour-bearing mice (*N*=5) were co-administered Doxil (10 mg kg^−1^) and ^89^Zr-NRep (8.0 mCi kg^−1^, Supplementary Table 2), and underwent *in vivo* PET imaging 24 h post-injection. Figure 2a shows two mice with vastly different ^89^Zr-NRep tumour uptake. Following the imaging session the anaesthetized mice were euthanized, after which digested tumours' DOX content was determined spectrofluometrically, while radioactivity was quantified by gamma counting. In line with the data presented in Fig. 1f, we found a strong correlation (*r*=0.97, *P*<0.01 (Pearson)) between uptake values determined by PET and DOX levels in tumours (Fig. 2b), a result we corroborated by *ex vivo* gamma counting (Fig. 2c). The linear dependency of ^89^Zr-PET signal and DOX delivery allowed us to create a calibration curve and, resulting from this, a method to non-invasively derive the amount of DOX accumulated in tumours.

### ^89^Zr-NRep is applicable to other nanoparticle platforms

**Figure 3:**
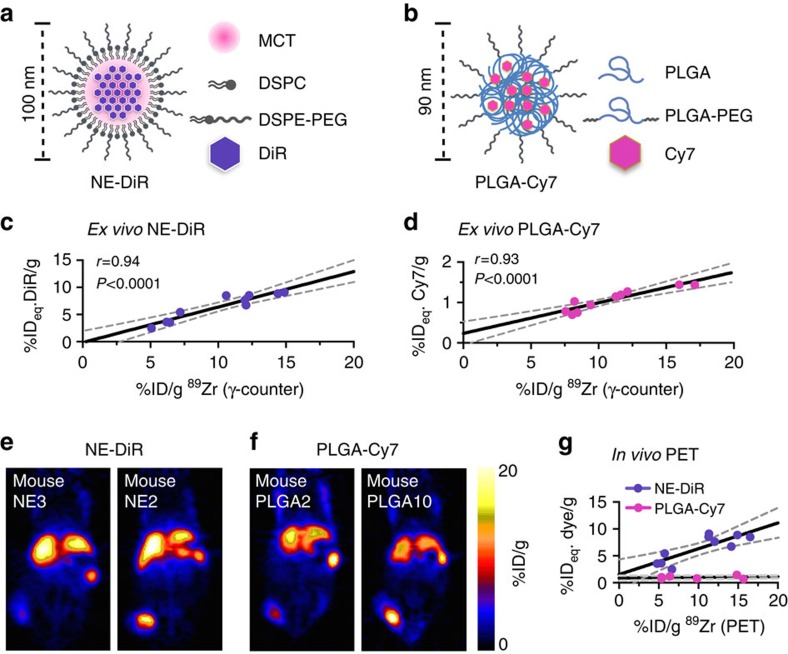
Nanoreporter PET quantifies nanoemulsion and PLGA nanoparticle uptake. Structure and composition of (**a**) NE-DiR and (**b**) PLGA-Cy7. Correlation between (**c**) ^89^Zr-NRep (%ID per g) and DiR (%ID_eq._/g) uptake (*N*=10), and (**d**) ^89^Zr-NRep (%ID per g) and Cy7 (%ID_eq._/g) uptake (*N*=10), at 24 h post co-administration of NE-DiR and ^89^Zr-NRep and PLGA-Cy7 and ^89^Zr-NRep, respectively. (**e**) Representative PET images of 4T1 tumour-bearing mice at 24 h post co-injection of NE-DiR and ^89^Zr-NRep showing low (mouse NE3, left) and high (mouse NE2, right) ^89^Zr uptake. (**f**) Representative PET images of 4T1 tumour-bearing mice at 24 h post co-injection of PLGA-Cy7 and ^89^Zr-NRep showing low (mouse PLGA2, left) and high (mouse PLGA10, right) ^89^Zr uptake. (**g**) Correlation between ^89^Zr uptake values generated by non-invasive PET imaging and DiR (purple (*N*=10), *r*=0.83, *P*<0.01) and Cy7 (pink (*N*=10), *r*=0.33, *P*=0.38) concentrations in tumours from mice co-injected with NE-DiR and ^89^Zr-NRep, and PLGA-Cy7 and ^89^Zr-NRep, respectively. Pearson's *r* coefficients were calculated to determine correlation.

Similar to what we showed for Doxil, we tested the ability of ^89^Zr-NRep to quantify the uptake of other non-liposomal nanoparticle systems. For this, we loaded a nanoemulsion with the fluorescent molecule DiR, yielding DiR-loaded nanoemulsion (Fig. 3a). We also tested a PLGA block copolymer nanoparticle, loaded with the fluorescent dye Cy7 (PLGA-Cy7, Fig. 3b). Analogous to what was done for ^89^Zr-NRep/DOX, we determined the correlation between ^89^Zr uptake by *ex vivo* γ-counting as well as non-invasive *in vivo* PET imaging and fluorophore accumulation by *ex vivo* spectrophotometry. The experiments were performed in a 4T1 breast cancer model (*N*=10 for each nanoparticle). Co-injection of the long-circulating nanoparticle systems (10 mg dye per kg for both NE-DiR (DiR-loaded nanoemulsion) and PLGA-Cy7) and ^89^Zr-NRep (8.0 mCi kg^−1^) resulted in a strong correlation between the radioactivity and fluorescence 24 h post administration (*r*=0.94 and 0.93 for NE-DiR and, PLGA-Cy7, respectively, Fig. 3c,d). The fitted line for PLGA-Cy7 intersects the *y* axis at 0.24±0.13 %ID_eq._ Cy7/g. This might be due to a tendency of PLGA-Cy7 to release its cargo before extravasation into the tumour, reducing the enhanced permeability and retention (EPR) accumulation component of the nanoformulation. PET scans showed vastly different ^89^Zr-Nrep uptakes (Fig. 3e,f), which correlated best with NE-DiR accumulation (Fig. 3g). While correlation for PLGA-Cy7 was strong *ex vivo* (Fig. 3d), PET data from the same mice did not yield a statistically significant correlation *in vivo*. This is not necessarily a shortcoming of the ^89^Zr-Nrep imaging strategy, but rather a result of the lower PLGA-Cy7 uptake in tumours when compared with Doxil and NE-DiR.

It is worth noting that we used nanoparticles that exhibit varying tumour accumulation profiles: clinically optimized Doxil accumulates to the highest extent; the nanoemulsion exhibits intermediate tumour accumulation; and the PLGA nanoparticle used for this study displayed the lowest tumour accumulation. Importantly, irrespective of their absolute tumour accumulation, the correlation with ^89^Zr-NRep is very strong (*r*>0.9) for all nanoparticles. This implies that in tumours, or tumour regions, wherever ^89^Zr-NRep uptake is high, relative nanoparticle uptake is high.

^89^Zr-NRep works particularly well for PEGylated long-circulating nanomaterials. We did, however, also evaluate its ability to monitor the protein-based nanoparticle albumin-bound paclitaxel (Nab paclitaxel, Abraxane). Although a strong correlation was observed (Supplementary Fig. 3), the plot does not intersect zero, indicative of Abraxane's ‘background' uptake independent of the EPR effect. In fact, Abraxane nanoparticles rapidly dissociate in the bloodstream, generating albumin fragments^17^. Therefore, the ^89^Zr-NRep technology can be best applied for a variety of nanoparticle platforms whose tumour accumulation is mainly dictated by the enhanced permeability and retention effect.

### ^89^Zr-NRep reveals Doxil's tumour uptake to vary vastly

**Figure 4:**
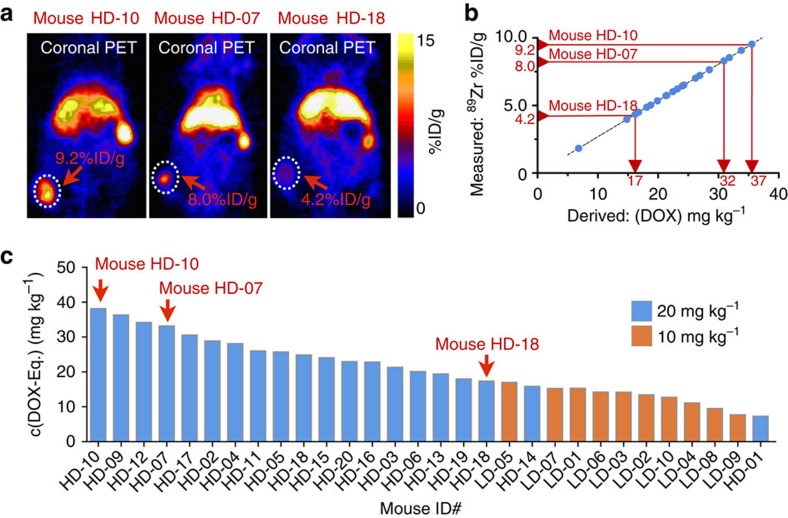
Quantifying DOX in individual mouse tumours by ^89^Zr-NRep PET imaging. (**a**) PET scans of mice HD-10 (large tumour, high uptake), HD-07 (small tumour, high uptake) and HD-18 (medium-sized tumour, low uptake), demonstrating intertumoural uptake heterogeneity. (**b**) Determination of intratumoural DOX concentrations based on ^89^Zr-NRep %ID per g uptake values. Only the data for animals injected with 20 mg kg^−1^ are shown. The data corresponding to the 10 mg kg^−1^ group are shown in Supplementary Fig. 5a. (**c**) Individual non-invasively determined intratumoural DOX concentrations for mice receiving either 20 mg kg^−1^ Doxil (*N*=20) or 10 mg kg^−1^ Doxil (*N*=10). Labelled red arrows in **b** and **c** indicate data points for mice HD-10, HD-07 and HD-18. Their PET scans are shown in **a**.

Encouraged by the robustness of our Doxil nanoreporter technology, we evaluated its applicability for treatment prognosis through an extensive therapeutic study. Breast cancer tumour-bearing mice (*N*=55) were randomly assigned to three different groups: saline control, ^89^Zr-NRep control and Doxil/^89^Zr-NRep treatment groups dosed at either 10 or 20 mg DOX per kg. At the start of treatment, the tumour sizes among the groups were similar (Supplementary Fig. 4a). Anaesthetized mice from the Doxil/^89^Zr-NRep groups underwent a PET imaging session 24 h post-injection. After this single imaging session, we used a caliper to measure tumour size in all groups three times per week until the animals were killed according to defined end points (Methods section). The different groups' tumour growth profiles are shown in Supplementary Fig. 4a–c. In comparing the ^89^Zr-NRep-injected group and saline-treated controls, we observed that ^89^Zr-NRep alone did not affect either tumour growth or survival rates (Supplementary Fig. 4). The animals that received Doxil, on the other hand, showed inhibited tumour growth rates and extended survival (Supplementary Fig. 4c,d). We also noted a dose effect between the high- and low-dose Doxil groups. Finally, by day 12 after treatment administration, the Doxil groups' tumour growth rates approximated those of the untreated groups, thereby indicating that Doxil's therapeutic effects had worn off (Supplementary Fig. 4a–c). Subsequent analyses of the *in vivo*^89^Zr-NRep PET data revealed highly varied DOX accumulation in animals at both Doxil dosage levels (10 and 20 mg kg^−1^). The range of ^89^Zr-NRep uptake heterogeneity can be seen in PET images from three different mice (Fig. 4a) showing high uptake in a large tumour (mouse HD-10), high uptake in a small tumour (mouse HD-07) and low uptake in a large tumour (mouse HD-18). Through interpolation of the PET-determined ^89^Zr uptake, using the calibration curve presented in Fig. 2, we were able to non-invasively derive individual intratumoural DOX concentrations (Fig. 4b, Supplementary Fig. 5a). We found DOX tumour concentrations ranging from 7.1 mg kg^−1^ all the way up to 36.8 mg kg^−1^ (Fig. 4c), with clear indication that animals receiving the lower dose had lower DOX accumulations in their tumours. Intriguingly, we found no correlation between tumour volume and ^89^Zr-NRep uptake (*r*=0.21, Supplementary Fig. 5b); tumour size, it seems, does not determine nanotherapeutic penetration.

### Predicting therapeutic outcome with ^89^Zr-NRep PET

**Figure 5:**
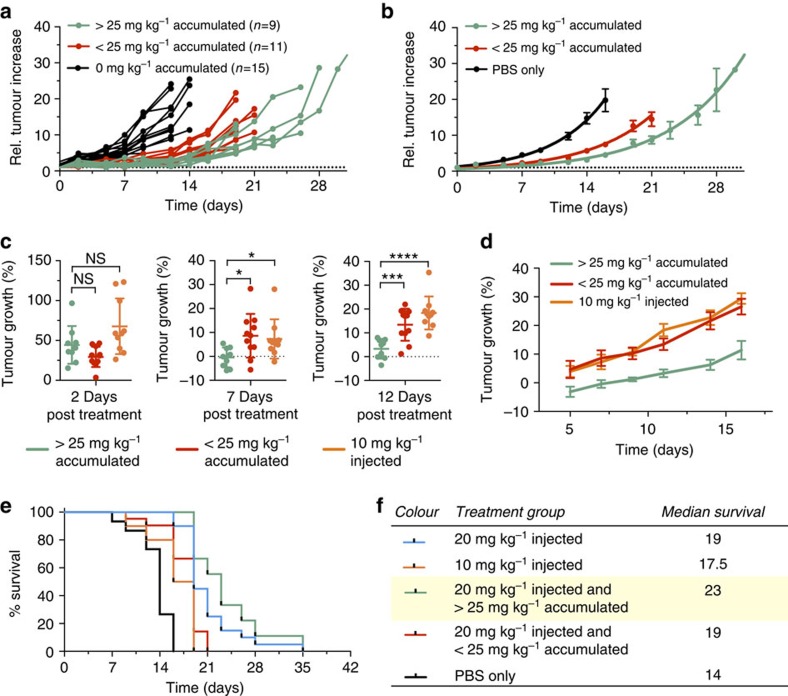
Predictive value of ^89^Zr-NRep PET imaging. (**a**) Individual tumour size increase in mouse cohorts treated with 20 mg kg^−1^ Doxil and >25 mg kg^−1^ intratumoural DOX concentration (*N*=9, green);<25 mg kg^−1^ intratumoural Doxil concentration (*N*=11, red) and controls (*N*=15, black). (**b**) Mean values of the groups in **a**. (**c**) Compared tumour growth rates for mice treated with 20 mg kg^−1^ Doxil that received >25 mg kg^−1^ intratumoural DOX (green, *N*=9), <25 mg kg^−1^ DOX (red, *N*=11) or 10 mg kg^−1^ Doxil (orange, *N*=10) at 2 days (left), 7 days (middle) and 12 days (right) post-treatment. The data from 2 days represent the initial daily growth rate (days 0–2, left); the 7- and 12-day data are the average daily growth rates from day 2 onwards. (**d**) Mean values of the average daily growth rates from day 2 onwards. (**e**,**f**) Kaplan–Meier plot and table showing the survival and median survival of individual mouse cohorts treated with 20 mg kg^−1^ Doxil (blue, *N*=20), 10 mg kg^−1^ Doxil (orange, *N*=10), 20 mg kg^−1^ Doxil and >25 mg intratumoural DOX per kg (green, *N*=9) or <25 mg intratumoural DOX per kg (red, *N*=11), plus the PBS treated control group (black, *N*=15). Error bars are s.e.m. *P* values were calculated with one-way analysis of variance (ANOVA) followed by Tukey's HSD; NS, not significant, *P<0.05, **P<0.01, ****P<0.0001.

Further retrospective investigation into relative tumour growth rates revealed distinct differences between individual mice that received no Doxil and mice that—based on *in vivo*^89^Zr-NRep PET—had either less or more than 25 mg kg^−1^ DOX accumulation in their tumours (Fig. 5a). Based on this observation, we subdivided the individual animals into three groups: controls, <25 mg kg^−1^ DOX or >25 mg kg^−1^ DOX (Fig. 5b). For ensuing analysis we also included the group treated with 10 mg kg^−1^ Doxil. Two days after Doxil administration and 1 day after the ^89^Zr-NRep PET scan, we saw no significant differences in percentage tumour growth among the different groups (Fig. 5c). Once therapeutic effects became appreciable (Supplementary Fig. 4c), we determined average growth rates from that time onwards. At day 7, the >25 mg kg^−1^ group had significantly slower tumour growth than the <25 mg kg^−1^ group (*P*<0.05 (one-way analysis of variance)). At day 12 the differences were even more statistically significant (*P*<0.01 and *P*<0.0001, respectively (one-way analysis of variance); Fig. 5c,d). All our analyses showed the same pattern (Supplementary Fig. 6): the initial lack of correlation and slow change towards significant correlations by day 7 of treatment effect. This result indicates that, facilitated by ^89^Zr-NRep PET, tumour growth inhibition can be predicted, thereby allowing retrospective re-categorization using DOX tumour content, measured *in vivo*, to increase intragroup homogeneity. For individual subjects, the initial ^89^Zr-NRep PET-derived uptake values serve as an inclusion criterion and robust treatment efficacy indicator. Finally, we investigated ^89^Zr-NRep PET's prognostic value. Using the animal subdivisions described above, we plotted survival in Kaplan–Meier curves (Fig. 5e). As with tumour growth, we observed increasingly enhanced survival among, in ascending order, control mice, mice treated with 10 mg kg^−1^ Doxil and mice treated with 20 mg kg^−1^ Doxil. Median survival for the low- and high-dose Doxil groups is, respectively, 25 and 36% greater than for the control group (Fig. 5f). Subdividing the high-dose Doxil group showed significantly enhanced survival in mice with more than 25 mg kg^−1^ DOX accumulated in their tumours (*P*=0.0004 (log-rank Mantel–Cox)). Taking the delivered dose into account, the median survival of animals with more than 25 mg kg^−1^ DOX in their tumours is 64 % longer than the control group (*P*<0.0001, Fig. 5f (log-rank Mantel–Cox)).

## Discussion

We have shown the accurate determination of long-circulating nanoparticle tumour accumulation using our PET nanoreporter ^89^Zr-NRep. Importantly, for Doxil, a nanoliposomal formulation of DOX, the non-invasively obtained PET-based values were predictive of therapeutic efficacy. It has to be emphasized that the presented nanoreporter technology did not require modification of clinical grade Doxil, which was obtained through the hospital pharmacy. Generally, the presented strategy has several advantages over the creation of so-called theranostic nanoparticles, which contain both a therapeutic and a diagnostic^18^. First, it enables accurate tumour accumulation imaging of (Food and Drug Administration (FDA) approved) nanoparticle drugs without the need for their chemical modification, guaranteeing the preservation of integrity, clinical grade and therapeutic efficacy. Second, since the same nanoreporter can be used for multiple platforms, as shown in Fig. 3, only one non-therapeutic, truly diagnostic agent needs to be developed and approved. Third, the nanoreporter PET imaging technology may impact next generation nanotherapeutics as it enables a more accurate identification of amenable patients and the exclusion of subjects that will not benefit from nanoparticle therapy. This is not only beneficial to patients, but also facilitates nanoparticle drugs' development and clinical evaluation, as the technology helps homogenizing therapeutic outcomes. Mechanistically, this is because EPR-driven uptake is mostly non-discriminating, as evidenced by the linear relationship between ^89^Zr-NRep and the different types of non-targeted nanoformulations used (Figs 1f, 2a,b and 3c,d). The overall nanoparticle uptake itself, however, is governed by extravasation rate and integrated blood pool concentrations, affecting the slopes of the fitted lines. As a result, particles with high overall uptake have steeper slopes (Doxil:m=1.08, Fig. 1f) than intermediate uptake particles (NE-DiR:m=0.65, Fig. 3c) and low uptake particles (PLGA-Cy7:m=0.08, Fig. 3d).

Importantly, as PET imaging is a quantitative and highly sensitive imaging modality, only trace amounts of the ^89^Zr-based nanoreporter are required. Furthermore, since PET imaging is a hot spot technique^19^, it does not require the acquisition of pre- and post-contrast images, followed by the analysis of changes in signal intensity, while this is required for both MRI and computed tomography (CT)^20^. Not only is this time consuming but also very sensitive to inaccuracies as organ movement and image artifacts complicate the procedure. Although CT is an inherently quantitative imaging technique, it is severely hampered by poor detection sensitivity for exogenously administered agents, requiring unrealistically high dosages, effectively preventing translation^20^. MRI is significantly more sensitive than CT, but is still orders of magnitudes less sensitive than PET, necessitating the administration of high doses of iron oxide contrast agents. Currently, the only agent available is ferumoxytol, applied to treat iron-deficiency anaemia, and used off-label for MRI (ref. 21). In light of a recent FDA warning (http://www.fda.gov/Drugs/DrugSafety/ucm440138.htm), its routine clinical application to determine nanoparticle tumour uptake seems unlikely.

Although our nanoreporter has very similar physicochemical properties to Doxil, their pharmacokinetics were not identical. Still, a very strong correlation between DOX and ^89^Zr tumour accumulation was observed. This suggests that the nanoreporter technology can also be used in conjunction with other nanoliposomal anti-cancer therapies. Similarly, strong correlations between NE-DiR or PLGA-Cy7 and ^89^Zr-NRep were found, implying potential for other nanoparticle drug classes, like second generation controlled release systems^22^.

In summary, our nanoreporter PET imaging technology allows robust and accurate *in vivo* determination of Doxil targeting and DOX tumour content. Together, our data and analyses demonstrate that DOX tumour accumulation is an important predictor of breast cancer growth inhibition and a prognostic parameter for survival. More generally, nanoreporter PET can evaluate FDA-approved nanotherapeutics without compromising their clinical grade or therapeutic efficacy. PET's inherently high sensitivity requires only a small amount of nanoreporter and therefore would not have any implications on nanotherapy dosing in patients. Since the technology does not necessitate modifying the clinical grade product and delineates a robust inclusion criterion, translation is within reach, which will help rejuvenate oncological nanotherapy^23^.

## Methods

### Chemicals

Phospholipids were purchased from Avanti Polar Lipids (Alabaster, AL). 1-(4-Isothiocyanatophenyl)-3-[6,17-dihyroxy-7,10,18,21-tetraoxo-27-[N-acetylhydroxyla-mino) 6,11,17,22-tetraazaheptaeicosane]thiourea (DFO-*p*-NCS) was purchased from Macrocyclics (Dallas, TX). The fluorescent labels Cy7-NHS ester and 1,1′-Dioctadecyl-3,3,3′,3′-Tetramethylindotricarbocyanine Iodide (DiR) were purchased from Lumiprobe GMBH and ThermoFisher Scientific, respectively. Pegylated liposomal doxorubicin (Doxil) and nanoparticle albumin-bound paclitaxel (Abraxane) were acquired from the Memorial Hospital pharmacy. All other reagents were acquired from Sigma-Aldrich.

### Animal model

The mouse breast cancer cell line 4T1 was obtained from ATCC (Manassas, VA), tested for mycoplasma contamination and grown in DMEM with 4.5 g l^−1^ L-glucose, 10% (vol/vol) heat inactivated foetal bovine serum, 100 IU penicillin and 100 μg ml^−1^ streptomycin purchased from the culture media preparation facility at Memorial Sloan Kettering Cancer Center (MSKCC, New York, NY). Female homozygous athymic nude NCr mice were obtained from Taconic Laboratories (Hudson, NY). Xenograft injections were performed on mice (8–10 weeks old) anaesthetized with 1–2% isoflurane (Baxter Healthcare, Deerfield, IL) in 2 l min^−1^ medical air. 4T1 cells were injected (1 × 10^6^ cells in 100 μl DMEM) subcutaneously, and the tumours grown for 7 days.

### Animal care

For all intravenous injections, mice were gently warmed with a heat lamp and placed on a restrainer. Their tails were sterilized with alcohol pads, and injections were placed into the lateral tail vein. All animal experiments were done in accordance with protocols approved by the Institutional Animal Care and Use Committee of MSKCC following National Institutes of Health guidelines for animal welfare.

### Synthesis of the phospholipid-chelator DSPE-DFO

The phospholipid-chelator DSPE-DFO was prepared according to our previously described procedure^24^. Briefly, DSPE (1,2-distearoyl-*sn*-glycero-3-phosphoethanolamine) and 1-(4-Isothiocyana-tophenyl)-3-[6,17-dihyroxy-7,10,18,21-tetraoxo-27-[N-acetylhydro-xylamino)-6,11,17,22-tetraazaheptaeicosane]thiourea (DFO-*p*-NCS) were reacted in a 1:1 dimethylsulfoxide/chloroform mixture in the presence of diethyl isopropylaimne at 50 °C for 48 h under nitrogen atmosphere. After cooling down to room temperature, chloroform was evaporated and water was added along with a 1-M Tris solution. The mixture was stirred for 30 min and filtered. The solid was washed with 1 M Tris, water and dichloromethane to produce the desired compound as a white solid in 70–80% yield.

### Liposome preparation

Liposomes were prepared using the sonication method, as previously reported^24^. Briefly, a lipid film was prepared by evaporating a chloroform solution containing the corresponding lipids in the desired proportion (Supplementary Table 1). The resulting film was hydrated with PBS (typically 10 ml) and sonicated for 25 min using a 150 V/T Ultrasonic Homogenizer (Biologics, Inc., Ramsey, NJ) working at 30% power output. After quick centrifugation, size and Z-potential measurements were performed on a NanoSeries Z-Sizer (Malvern Instruments, Malvern, UK) and a Zeta PALS analyser (Brookhaven Instruments Corporation, Holtsville, NY), respectively. Liposomes containing DSPE-DFO were concentrated using a 100-kDa VivaSpin (Millipore, Billerica, MA) tube and washed twice with PBS.

### Synthesis of the Cy7-loaded PLGA-PEG nanoparticles Cy7-PLGA

A lipophilic fluorescent Cy7 analogue was synthesized via conjugation of Cyanine7-N-Hydroxysuccinimide (NHS) ester and oleylamine. The NHS ester and the amine (1:1.1 molar ratio) were reacted in anhydrous dichloromethane in the presence of triethylamine. The mixture was stirred in the dark at room temperature for 20 h, and the reaction was monitored by thin layer chromatography (chloroform/methanol, 5:1, v/v). The solvent was then removed under reduced pressure, and the residue was redissolved in acetonitrile. The unreacted amine was removed by filtration. The identity of the product was confirmed by mass spectrometry.

Self-assembled Cy7 nanoparticles were synthesized by the nanoprecipitaion method. Poly(ethylene glycol) methyl ether-block-poly(lactide-co-glycolide; pegylated poly(lactic-co-glycolic acid) (PLGA-PEG); PLGA Mn 4000, PEG Mn 2000; 200 mg), PLGA (lactide:glycolide, 50:50, Mw 30–60 k; 84 mg), and C_18_-Cy7 (2 mg) were dissolved in acetonitrile at a concentration of 10 mg ml^−1^. To form nanoparticles, the acetonitrile solution was dripped into 20 ml PBS at a rate of 0.2 ml min^−1^ at room temperature under vigorous stirring. The solution was then continuously stirred for 1 h after dripping to induce evaporation of the organic solvent. The resulting nanoparticles were purified through a first centrifugation at 18 g for 10 min to remove possible aggregates, and then washed at least three times with fresh PBS using 100 molecular weight cut-off (MWCO) centrifugal filters and concentrated. Nanoparticles were kept at 4 °C and protected from light until use. The concentration of dye in the nanoparticle solution was determined spectrofluorimetrically (*λ*_Ex_=750 nm, *λ*_Em_=775 nm). The size of the particles, as determined by dynamic light scattering (DLS), was 93.7±0.5 d.nm, polydispersity index (PDI): 0.16±0.01.

### Synthesis of the DiR-loaded nanoemulsion DiR-NE

A nanoemulsion containing the lypophilic dye DiR was synthesized by the diffusion method. A stock solution of mixed lipids was prepared in ethanol at 25 mg ml^−1^, containing 1,2-distearoyl-*sn*-glycero-3-phosphocholine (DSPC), cholesterol and DSPE-PEG2000 (62:33:5 mole ratio). Middle chain triglycerides and DiR were added to this solution at a weight ratio of lipids:middle chain triglycerides:DiR=1:2:0.01. The nanoemulsion was prepared by swiftly injecting 1 ml of this ethanolic mixture into 20 ml of PBS under vigorous stirring. The resulting nanoemulsion was purified through a first centrifugation at 18 g for 10 min to remove possible aggregates, and then washed at least three times with fresh PBS using 100 kDa MWCO centrifugal concentrators and finally concentrated. The concentration of DiR in the nanoemulsion solution was determined by spectrofluorimetry (*λ*_Ex_=750 nm, *λ*_Em_=775 nm). The size of the particles, as determined by DLS, was 99.0±0.9 d.nm, PDI: 0.15±0.01.

### Preparation of Cy5.5-albumin@Abraxane

Cy5.5-albumin was prepared by reacting albumin and Cy5.5 NHS ester in PBS buffer pH 7.6–8.0 for 16 h at room temperature. Cy5.5 NHS ester was added as a DMSO solution to achieve a 2:1 mol ratio over albumin. The fluorescent protein was purified by spin filtration using 10 kDa MWCO tubes at 4,000 r.p.m., and washed twice with PBS. The retentate was finally diluted to a final concentration of 2 mg ml^−1^ with sterile saline solution and filtered through a 0.22-μm filter before use.

Fluorescent Abraxane nanoparticles were prepared by mixing Cy5.5-albumin (380 μl, 0.76 mg) and 300 μl of reconstituted Abraxane formulation in saline containing 3.0 mg paclitaxel and 27 mg albumin, thus making Cy5.5-albumin <3% of total albumin. The sample was left at 4 °C for 24 h to allow equilibration.

### Radiochemistry

^89^Zr was produced at Memorial Sloan Kettering Cancer Center on an EBCO TR19/9 variable-beam energy cyclotron (Ebco Industries Inc., BC, Canada) via the ^89^Y(p,n)^89^Zr reaction and purified in accordance with previously reported methods to yield ^89^Zr with a specific activity of 195−497 MBq μg^−1^ (ref. 25). Activity measurements were made using a Capintec CRC-15R Dose Calibrator (Capintec, Ramsey, NJ).

### HPLC and Radio-HPLC

High-performance liquid chromatography (HPLC) was performed on a Shimadzu HPLC system equipped with two LC-10AT pumps and an SPD-M10AVP photodiode array detector. Radio-HPLC was performed using a Lablogic Scan-RAM Radio-TLC/HPLC detector. Size exclusion chromatography was performed on a Superdex 10/300 column (GE Healthcare Life Sciences, Pittsburg, PA) using PBS as eluent at a flow rate of 1 ml min^−1^.

### Radiosynthesis of ^89^Zr-nanoreporter (^89^Zr-NRep)

A solution of 0.3 % DFO-bearing liposomes in PBS was reacted with ^89^Zr-oxalate at 40 °C for 2 h (ref. 24). The labelled liposomes were separated from free unreacted ^89^Zr by spin filtration using 100 kDa molecular weight cutoff tubes (Millipore, Billerica, MA). The retentate was washed with sterile phosphate buffered saline (PBS, 3 × 0.5 ml) and finally diluted with sterile PBS to the desired volume. The radiochemical yield was 86±3% (*n*=6) and the radiochemical purity >99% (Fig. 1c).

### Determination of radioactivity content and doxorubicin concentration in tumours

Female homozygous athymic nude NCr mice (*N*=24) bearing 4T1 tumours grown over 7 days were injected with a mixed dose containing Doxil (10 mg doxorubicin per kg body weight) and ^89^Zr-NRep (20.3±3.9 μCi, Supplementary Table 2). At predetermined time points (6, 24 and 48 h), animals were killed and perfused with PBS. Tumours were collected and weighed. Larger tumours were divided into portions of ∼50 mg. The resulting tumour samples were counted using a Wizard2 2480 Automatic Gamma Counter (Perkin Elmer, Waltham, MA). The doxorubicin concentration was quantified as previously reported^26^. Briefly, immediately after gamma counting, tumour samples were homogenized in lysis buffer (10:1 v/w ratio) using a hand-held electrical homogenizer. Aliquots of 200 μl of homogenate were transferred to a new tube, and water (200 μl), Triton X-100 (10 % solution in water, 100 μl) and finally acidified isopropanol (0.75 N HCl, 1.5 ml) were added. The mixture was vortex mixed and left at −20 °C for 16 h. Samples were then vortexed, centrifuged at 15,000 r.p.m. for 20 min, and aliquots of 200 μl were measured on a 96-well plate using a Safire microplate reader (Tecan, Männedorf, Switzerland). A calibration curve was generated by adding increasing amounts of doxorubicin to tumour sample homogenates (prepared as described above) from animals not treated with Doxil.

### PET imaging

Female homozygous athymic nude NCr mice (*N*=35) bearing 4T1 breast tumours were injected with 0.14–0.17 mCi ^89^Zr-NRep mixed with the corresponding dose of Doxil (see Supplementary Table 2 for detailed composition). At 24 h the animals were anaesthetized with isoflurane/oxygen gas mixture (2% for induction, 1 % for maintenance), and a scan was then performed using a Focus 120 microPET scanner (Siemens Medical Solutions, Inc., Malvern, PA). Whole-body PET static scans recording a minimum of 20 million coincident events were performed, with durations of 10–15 min The energy and coincidence timing windows were 350−700 keV and 6 ns, respectively. The image data were normalized to correct for non-uniform responses to PET, dead-time count losses, positron branching ratio and physical decay to the time of injection, but no attenuation, scatter or partial-volume averaging correction was applied. The counting rates in the reconstructed images were converted to activity concentrations (percentage injected dose (%ID) per gram of tissue) using a system calibration factor derived from imaging a mouse-sized water-equivalent phantom containing ^89^Zr. Images were analysed using ASIPro VMTM software (Concorde Microsystems, Knoxville, TN). Activity concentration was quantified at the end of the study by averaging the maximum values in at least 5 region of interests drawn on adjacent slices of tumour tissue^27^.

### ^89^Zr-NRep as nanoreporter for Cy-PLGA and DiR-NE nanoparticles

Female homozygous athymic nude NCr mice (8–10 weeks old, *N*=20) bearing 4T1 breast tumours grown over 8 days were co-injected with Cy7-PLGA (10 mg Cy7 per kg, *N*=10) or DiR-NE (10 mg DiR per kg, *N*=10), and ^89^Zr-NRep (8.0 mCi kg^−1^, 0.17–0.18 mCi). The nanomaterials were allowed to circulate for 24 h, after which time a PET scan was performed as described. The mice were then killed and perfused with PBS. Tumours were collected, blotted and weighed before radioactivity counting. Cy7 and DiR concentration in tumours was determined by spectrofluorimetry as described for DOX (see ‘Determination of radioactivity content and doxorubicin concentration in tumours') using acetonitrile instead of 0.75 N HCl isopropanol for extraction of the dyes, and excitation and emission wavelengths were 750 and 775 nm, respectively.

### ^89^Zr-NRep as nanoreporter for Abraxane

Female NCr nude mice bearing 4T1 breast cancer tumours (*N*=6) grown for 7 days were administered a single dose containing Cy5.5-albumin@Abraxane (25 mg PCT per kg) and ^89^Zr-NRep (5.0 mCi kg^−1^) via the lateral tail vein. Twenty four hours after administration, animals were killed and perfused. Tumours were collected and weighed, and their radioactivity content was measured by gamma counting. Immediately after counting, tumours were homogenized in lysis buffer added at a 10:1 volume/weight ratio. A 200-μl aliquot was transferred to another gamma counter tube and Triton X-100 (100 μl, 10 % solution in water) and water (700 μl) were added. The mixture was vortexed and left at 4 °C for 24 h. Samples were then vortexed, centrifuged at 15,000 r.p.m. for 20 min and aliquots of 200 μl were measured spectroflurimetrically on a 96-well plate using 675 and 705 nm as excitation and emission wavelengths, respectively. A calibration curve was generated by adding increasing amounts of Cy5.5-albumin to tumour homogenates obtained from untreated animals.

### Therapeutic study

Female homozygous athymic nude NCr mice (8–10 weeks old, *N*=55) bearing 4T1 breast tumours grown over 7 days were divided into three groups receiving saline (PBS, *N*=15), ^89^Zr-NRep (*N*=10) and Doxil/^89^Zr-NRep (*N*=30). The last group was divided in two subgroups according to the DOX dose injected (10 or 20 mg kg^−1^, *N*=10 and 20, respectively). Dose composition can be found in Supplementary Table 2. The doses were administered via the lateral tail vein. The treatment groups (low and high DOX) had a PET imaging scan 24 h post-injection. Control groups were anaesthetized for the duration of a typical PET scan (10–15 min) as a mock imaging session. All groups were monitored for tumour size three times weekly using digital calipers to take the longest (*L*) and shortest (*S*) perpendicular diameters. The volume was calculated using the formula *V*=(*L*x*S*^2^)/2 (ref. 28). Euthanasia was scheduled according to predetermined end points: either a tumour volume >600 mm^3^ or notification by the Research Animal Resource Center personnel from Memorial Sloan Kettering Cancer Center. After the end of the study, PET images were analysed as described above to determine uptake values.

### Statistical analysis

Data are expressed as mean±s.d. or s.e.m. Data were analysed using one-way variance analysis, followed by Tukey's honest significant different test. Pearson's *r* coefficients were calculated to determine correlation. Log-rank Mantel–Cox tests were performed for survival analysis. Statistical analyses were performed with GraphPad Prism, Version 6.0c (La Jolla, CA) and *P* values<0.05 were considered significant.

### Data availability

All relevant data are available from the authors.

## Additional information

**How to cite this article:** Pérez-Medina, C. *et al.* Nanoreporter PET predicts the efficacy of anti-cancer nanotherapy. *Nat. Commun.* 7:11838 doi: 10.1038/ncomms11838 (2016).

## Supplementary Material

Supplementary Information Supplementary Figures 1-6 and Supplementary Tables 1-2
