# Supplementary material for: Nanoreporter PET predicts the efficacy of anti-cancer nanotherapy
Source: Nat Commun. 2016 Jun 20;7:11838. doi: 10.1038/ncomms11838 (PMC4915130; doi:10.1038/ncomms11838)
Supplement: Supplementary Information — Supplementary Figures 1-6 and Supplementary Tables 1-2 [file ncomms11838-s1.pdf]

SUPPLEMENTARY INFORMATION

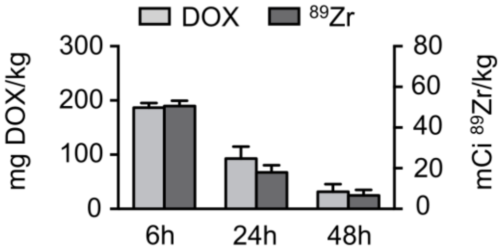

Supplementary Fig. 1 | ^{89}Zr-NRep mirrors DOX concentration in the blood pool. Absolute blood pool concentrations for DOX and ^{89}Zr (at 6h, 24h and 48h post injection) in mg DOX/kg and mCi ^{89}Zr/kg, respectively, in mice co-injected with Doxil (at 20 mg DOX/kg) and ^{89}Zr-NRep (at 8.0 mCi/kg). DOX and radioactivity concentrations were determined using the same procedure as with tumor samples.

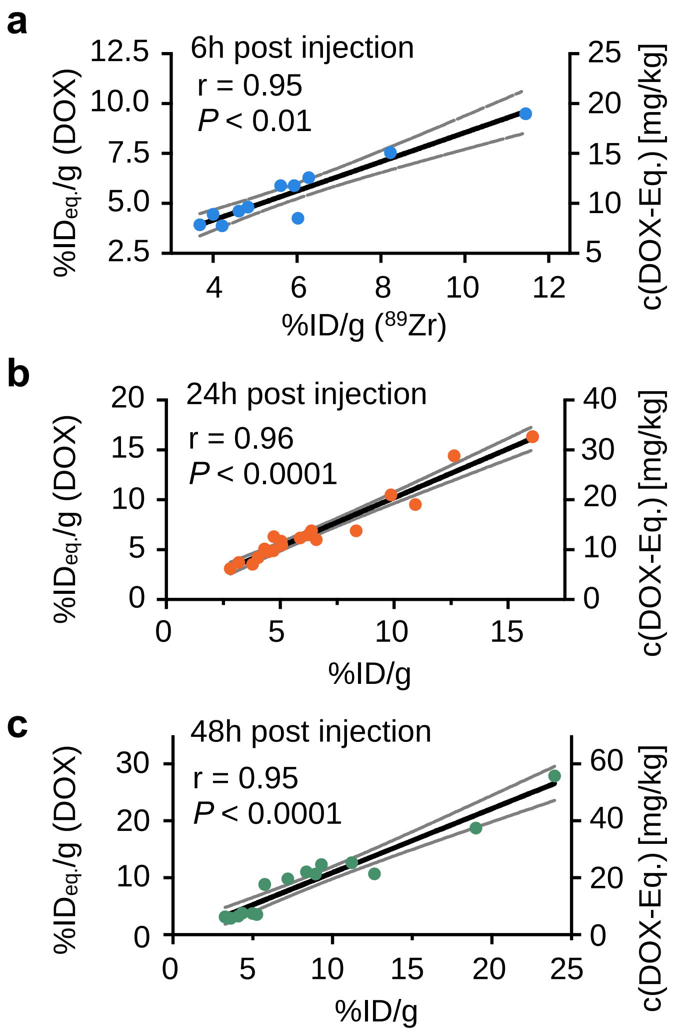

Supplementary Fig. 2 | ^{89}Zr-NRep mirrors DOX accumulation in tumor. Curves show correlation between radioactivity and doxorubicin fluorescence determined ex vivo in digested tumor samples at (**a**) 6 h, (**b**) 24 h and (**c**) 48 h after co-injection of Doxil and ^{89}Zr-NRep. Pearson's r coefficients were calculated to determine correlation.

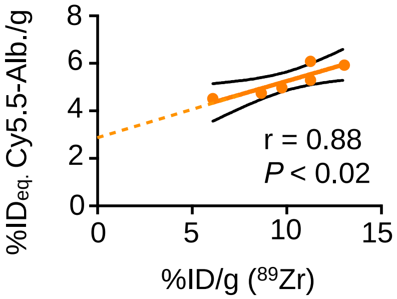

Supplementary Fig. 3 | ^{89}Zr-NRep as nanoreporter for Abraxane®. Correlation between ^{89}Zr-NRep (%ID/g) and fluorescently labeled Cy5.5-Albumin@Abraxane (%$ID_{eq.}$/g), determined *ex vivo* in digested tumor samples (N = 6) at 24 h after co-injection of the nanomaterial and ^{89}Zr-NRep. Pearson's r coefficient was calculated to determine correlation.

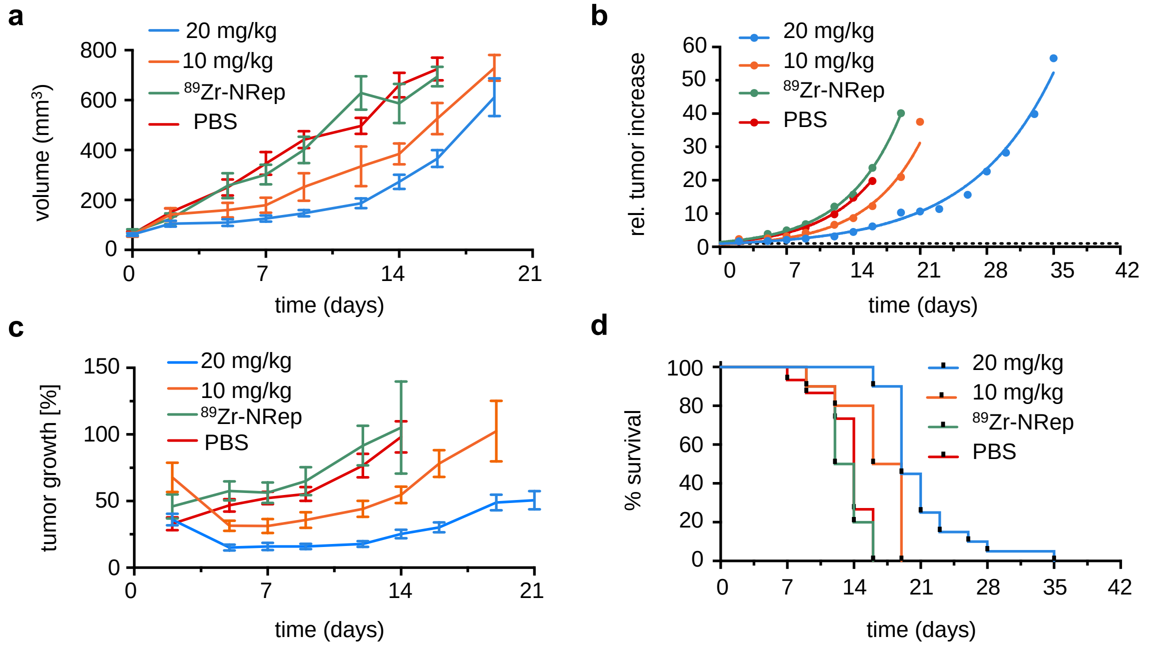

Supplementary Fig. 4 | Tumor growth for the different groups of 4T1 tumor-bearing female NCr nude mice used in the therapeutic study. (**a**) Tumor volume (mm^3) vs. time (days post injection). (**b**) Relative tumor increase after administration of the corresponding doses. (**c**) Average cumulative daily tumor growth rates. (**d**) Survival curves for the different groups in the therapeutic study. Data are expressed as mean ± s.e.m.

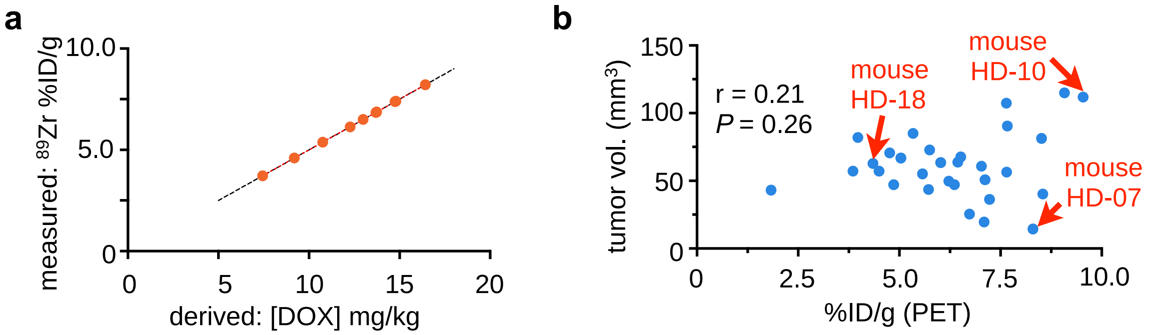

Supplementary Fig. 5 | ^{89}Zr-NRep uptake does not correlate with tumor size. (**a**) Uptake values obtained for Doxil treatment mice (N = 30) versus tumor volumes at time of administration. Labeled red arrows indicate data points for mice HD-10 (large tumor, high uptake), HD-07 (small tumor, high uptake) and HD-18 (medium-sized tumor, low uptake). Their PET scans and uptake values are shown in figure 3. (**b**) Determination of intratumoral DOX concentrations based on ^{89}Zr-NRep %ID/g uptake values, data shown for mice receiving 10 mg/kg Doxil (N = 10).

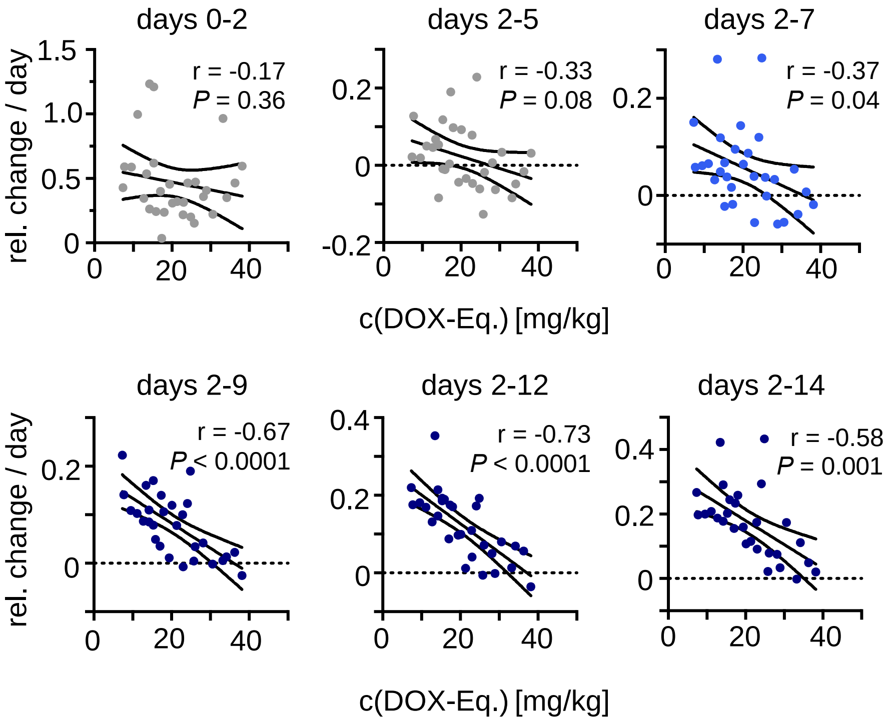

Supplementary Fig. 6 | DOX concentration in tumors, as determined by ^{89}Zr-NRep PET imaging, inversely correlates with tumor growth rate. Significant inverse correlations between the non-invasively determined amounts of DOX delivered to tumors can be observed at 7, 9, 12 and 14 days post administration. Pearson's r coefficients were calculated to determine correlation.

Supplementary Table 1. Lipid composition (in mol %), size (as mean effective diameter, MED) polydispersity index (PDI) and Z-potential of the different liposomes used.

	DPPC*	Cholesterol	DSPE-PEG2000	DSPE-DFO	MED / nm	PDI	Z-Potential / mV
**Doxil**	53	42	5	-	82.4 ± 0.2	0.05 ± 0.01	-31.1 ± 11.9
**Plain**	61.6	33.4	5	-	103.9 ± 0.7	0.11 ± 0.02	-23.7 ± 4.7
**^{89}Zr-NRep**	61.3	33.4	5	0.3	113.8 ± 3.1	0.15 ± 0.02	-26.1 ± 8.9**

* HSPC (hydrogenated soy phosphatidylcholine) for Doxil.
** Unlabeled.

Supplementary Table 2. Composition (expressed as μmol of total lipids, activity and mg of doxorubicin), size (MED) and polydispersity index (PDI) of the doses used in the study.

	Doxil	Plain	^{89}Zr-NRep / μCi	DOX	MED / nm	PDI
**Ex vivo**	2.4 μmol	-	20.3 ± 3.9	0.2 mg	83.0 ± 1.2	0.06 ± 0.01
**In vivo**	2.4 μmol	-	142 ± 1	0.2 mg	88.9 ± 0.7	0.10 ± 0.03
**^{89}Zr-NRep only**	-	4.8 μmol	163 ± 16	-	106.7 ± 1.6	0.12 ± 0.01
**Low Doxil**	2.4 μmol	2.4 μmol	163 ± 13	0.2 mg	94.1 ± 1.6	0.11 ± 0.01
**High Doxil**	4.8 μmol	-	165 ± 12	0.4 mg	87.4 ± 1.2	0.09 ± 0.01
